# Resourceful Event-Predictive Inference: The Nature of Cognitive Effort

**DOI:** 10.3389/fpsyg.2022.867328

**Published:** 2022-06-30

**Authors:** Martin V. Butz

**Affiliations:** ^1^Neuro-Cognitive Modeling Group, Department of Computer Science, University of Tübingen, Tubingen, Germany; ^2^Department of Psychology, Faculty of Science, University of Tübingen, Tubingen, Germany

**Keywords:** grounded cognition, cognitive resources, cognitive effort, predictive coding, active inference, event-predictive cognition

## Abstract

Pursuing a precise, focused train of thought requires cognitive effort. Even more effort is necessary when more alternatives need to be considered or when the imagined situation becomes more complex. Cognitive resources available to us limit the cognitive effort we can spend. In line with previous work, an information-theoretic, Bayesian brain approach to cognitive effort is pursued: to solve tasks in our environment, our brain needs to invest information, that is, negative entropy, to impose structure, or focus, away from a uniform structure or other task-incompatible, latent structures. To get a more complete formalization of cognitive effort, a resourceful event-predictive inference model (REPI) is introduced, which offers computational and algorithmic explanations about the latent structure of our generative models, the active inference dynamics that unfold within, and the cognitive effort required to steer the dynamics—to, for example, purposefully process sensory signals, decide on responses, and invoke their execution. REPI suggests that we invest cognitive resources to infer preparatory priors, activate responses, and anticipate action consequences. Due to our limited resources, though, the inference dynamics are prone to task-irrelevant distractions. For example, the task-irrelevant side of the imperative stimulus causes the Simon effect and, due to similar reasons, we fail to optimally switch between tasks. An actual model implementation simulates such task interactions and offers first estimates of the involved cognitive effort. The approach may be further studied and promises to offer deeper explanations about why we get quickly exhausted from multitasking, how we are influenced by irrelevant stimulus modalities, why we exhibit magnitude interference, and, during social interactions, why we often fail to take the perspective of others into account.

## 1. Introduction

Our cognitive abilities are limited. We are forgetful in so many ways, lazy, and often stuck with sub-optimal task solutions. Dual- or even multi-tasking is often extremely effortful and error-prone, unless one of the tasks can be done fully automatically. Why can't we do it? The answer is that our “cognitive resources” are limited, restricting the “cognitive effort” we can spend on the current tasks at hand. Some of us may be satisfied with such an answer. Clearly though, neither “cognitive” nor “resources” nor “effort” are really well-defined. This article attempts to scrutinize what “cognitive resources” and “cognitive effort” are.

Recent insights from cognitive science imply that our minds' thoughts—and thus our minds' mental content—while being awake, do not only cover the present itself, but also the past and future to varying extents (Buckner and Carroll, 2007; Butz et al., 2019, 2021; Stawarczyk et al., 2021). Meanwhile, these thoughts are structured in the form of event-predictive encodings (Rao and Ballard, 1999; Hommel et al., 2001; Zacks and Tversky, 2001; Zacks et al., 2007; Frings et al., 2020), giving rise to event-predictive cognition (EPCog, cf. Franklin et al., 2020; Zacks, 2020; Baldwin and Kosie, 2021; Butz et al., 2021; Kuperberg, 2021). EPCog essentially suggests that we hold thoughts about events, event progressions, and characteristics of the scenes within which these events unfold. When considering a particular event, we tend to temporally bind event-characterizing encodings of the entities, the interaction dynamics, and typical starting and ending conditions into an event-predictive attractor.

Meanwhile, we actively explore these events and progressions thereof by means of active inference (ActInf, cf. Friston, 2009; Friston et al., 2018), which formalizes (i) short-term state- and event-inference (inferring what is going on), (ii) goal-directed planning, reasoning, and behavioral control (inferring what to do), as well as (iii) longer-term, retrospective behavioral optimization and model learning (inferring useful memory structures). Derived from the free energy principle (Friston, 2009), ActInf, as a process theory, essentially formalizes the brain's effort to minimize the conjoint effort of brain and body to survive, that is, to maintain an inner, bodily-grounded balance, akin to a highly complex autopoietic system (Maturana and Varela, 1980; Butz, 2008; Friston et al., 2015b, 2018). In typical psychological paradigms, this effort may be closely related to a simple cost-benefit analysis to find solutions to particular tasks with minimum effort (Wang et al., 2021).

Given EPCog encodings, ActInf formalizes how these predictive encodings are dynamically activated and bound together. Current activation densities essentially encode the considered events and event progressions within partially defined scenes (Butz, 2016; Schrodt et al., 2017; Gumbsch et al., 2021a; Stegemann-Philipps and Butz, 2021; Achimova et al., 2022). As a result, ActInf offers a quantitative formalism to model the goal-oriented direction of attention, the dynamic maintenance of active working memory content, dynamic reasoning and decision making, and a purposeful selection, activation, and control of behavior (Gumbsch et al., 2021a,b). Conjoint with event-predictive cognition, ActInf flexibly binds components into Event-Gestalten, integrates them into scenes, projects them into potential futures, and controls their dynamic interactions in a goal-directed, homeostasis-oriented manner.

In accordance with these theories, cognitive effort may be formalized by means of a Resourceful Event-Predictive Inference (REPI) model. REPI is closely related to previous approaches that have equated cognitive effort with the information needed to change prior into posterior densities, given some source of information (Ortega and Braun, 2013; Genewein et al., 2015; Zenon et al., 2019). Beyond these previous approaches, though, REPI integrates event-predictive encoding structures into the formalism and offers an actual implementation of the inference dynamics. We thus can simulate several basic findings—such as the Simon effect and task switching costs—and offer suggestions for imminent model expansions. Moreover, we can measure the cognitive, task-oriented effort explicitly while the dynamics unfold. Dynamic processing in REPI is also similar to previous dynamic processing models, which did not explicitly relate the simualted processing dynamics to cognitive effort. For example, Steyvers et al. (2019) simulate dynamic task and response side densities. The model yields the basic task switching effect. Moreover, it can simulate training effects by increasing an adaption rate. Apart from the relation to cognitive effort, we simulate cognitive processing dynamics in the full processing pipeline, including stimulus and stimulus-bias estimation densities.

The remainder of this work details how this cognitive effort may be well-approximated by a mixture of computational-algorithmic processes, which control our thoughts. Thereby, the nature of the structures co-determines the resources needed to execute a particular task: the more compressed and readily available particular task-relevant structures are, the less cognitive resources will be required. In this paper, first more detailed background is provided on related work and on the main motivation for proposing REPI. Next, REPI is detailed on a computational level (Marr, 1982) and an algorithmic implementation is introduced. The system implementation generates behavioral results that yield the SIMON effect and task switching behavior. Other well-known psychological phenomena may be explained with REPI, including the STROOP, SNARC, and crossmodal interaction effects as well as cognitive limitations in social interactions. A final discussion concludes the work.

## 2. Information as Effort

The perspective that our mind and body somewhat invest information to survive dates rather far back. From the very general perspective of thermodynamics and quantum mechanics, Schroedinger (1944) already proposed that life itself may be characterized as the active intake of negative entropy, thus counteracting disintegration. Only over the last decade, however, several researchers have started formalizing how exactly our brains may invest information as a resource to elicit self-motivated, goal-directed decisions and behavior (Ortega and Braun, 2013; Genewein et al., 2015; Zenon et al., 2019).

### 2.1. Computational Information Processing Effort

The perspective that planning, reasoning, decision making, and control requires information processing resources can be partially motivated by, but it can also partially explain, the concept of bounded rationality (Bratman, 1987; Friston et al., 2013; Lieder and Griffiths, 2020). Bounded rationality essentially emphasizes that limited cognitive capacities prevent us from thinking problems fully through, deeming our decisions sub-optimal (Simon, 1955). To save resources, we employ heuristics and often act habitually rather than in a deliberate, goal-directed manner, which leads to typical errors in our reasoning and decision making (Kahneman, 2003; Gigerenzer and Gaissmaier, 2011). Thus, sub-optimal behavior is caused by our limited information processing resources, which struggle to process more complex tasks optimally due to their higher demand of information processing resources.

Ortega and Braun (2013) proposed a free energy-based formalization of bounded rational decision making that is directly derived from thermodynamics. Decision making requires to steer distributions away from a prior distribution toward a decision-peaked distribution. Changing the distribution requires information processing resources (e.g., to solve a task), while its investment is rectified by the corresponding expected utility gain (e.g., actually solving the task). Genewein et al. (2015) enhanced this model by enabling it to selectively recruit (provided) hierarchical encodings to further minimize cognitive effort. For example, the group of food items or dangerous animals is recruited, subsuming the entities that match with a respective interaction class, leading to a more efficient decision making process.

Focusing only on the cost of cognition—implicitly assuming that the effort will be worth the while and thus maximum available effort will be spent—(Zenon et al., 2019) formalize an optimization model that consists of three summands: the mutual information I between (i) the expected and actually perceived state of the environment, (ii) the expected prior (preparatory) response and actual posterior response, given the actually perceived state, and (iii) the assumed context *T* dependent stimulus-response mapping vs. the actual posterior response. The formalism thus assumes independency between stimulus processing effort, task-dependent response effort, and context-dependent response effort. The authors then illustratively address inferences in the Strop task and also discuss the consequences in task switching and multitasking, which we will follow up upon below.

REPI enhances the model of Zenon et al. (2019) and Genewein et al. (2015) in that it assumes an inner, event-predictive, generative model, which contains distributed predictive encoding densities. In line with Ortega and Braun (2013) and Zenon et al. (2019), REPI equates cognitive effort with the focused activation of current mental content away from both residual, previous task priors and latent, habitual priors. The mental content, however, is made more explicit and the active inference dynamics as well as the involved effort is simulated as a process model.

### 2.2. Neuro-Physiological Basis

While this paper does not address the question how cognitive effort is actually spent in the brain from a neuro-physiological perspective, it may generally be assumed that evolution has designed our brains to generate flexible and highly adaptive behavior—including socially-interactive behavior—in a very resource-effective manner. Accordingly, neuro-physiological evidence is accumulating that synchronized and coordinated neural firing is indeed effortful. It appears that processing resources are dominantly determined by the effort to generate and transmit signals via biochemical dynamics and neural spikes (Laughlin, 2001; Sengupta et al., 2010). Accordingly, energy is required for the maintenance of a wakeful state, in which neurons communicate with each other across cortical and sub-cortical areas by selective, rhythmic synchronization and coordination (Engel et al., 2001; Bastos et al., 2015; Fries, 2015; Daume et al., 2017; Misselhorn et al., 2019).

These and related insights are in agreement with the perspective that cognitive effort is needed to counter uncertainty, which, in neuro-physiological terms, is closely related to focusing the mind by means of precise, coordinated neural and biochemical activities (Daume et al., 2017; Misselhorn et al., 2019). Most recent research has even linked such resource constraints to predictive coding theory, showing that resource-efficient encodings are predictive and develop in recurrent neural networks naturally, when the goal is to solve a particular tasks resource-efficiently (Ali et al., 2021).

In sum, it may be said that the current's mind focus with its neuro-physiological activities essentially constitutes current mental content, Thereby, the maintenance of a stronger processing focus and dynamic refocusing is effortful. In order to make the mental content more explicit on a computational and algorithmic level, though, the active inference principle conjoint with event-predictive cognition offer suggestions.

## 3. Inference and Information Processing

To maximize adaptivity and flexibility, computational resources need to be directed toward those considerations that are, or may soon or suddenly become, behaviorally relevant (Butz et al., 2003; Butz, 2008; Butz and Pezzulo, 2008; Pezzulo et al., 2008). Various research strands have put forward that planning and reasoning may be viewed as inference processes, essentially subsuming model-free and model-based reinforcement learning, visual perception, and even social cognition (Rao and Ballard, 1999; Botvinick et al., 2009; Friston, 2009; Botvinick and Toussaint, 2012; Baker et al., 2017).

The involved perception, planning, and motor control mechanisms can be formalized by means of the active inference (ActInf) principle. Additionally, though, inference requires information processing resources, which is again intrinsically linked to ActInf.

### 3.1. Active Inference

ActInf essentially highlights that our brain predicts the future given its past experiences, even including its genetically-gathered experiences (Hohwy, 2013; Friston et al., 2015a; Clark, 2016). Importantly, though, our brain does not predict the future—or generate models of our environment—for its own sake. Rather, it does so for optimizing the generation of (highly adaptive) behavior, which is elicited to maintain and foster both internal homeostasis and model consistency (Friston et al., 2015b). As a result, the developing predictive encodings, which constitute our individual *latent* beliefs about the world, serve a self-motivated purpose. Together, our individual ActInf processes, which unfold within our individual predictive encoding structures, generate our individual current state of mind.

In relation to behavioral psychology, ActInf offers a formalization of the ideomotor principle: initial evolutionary-determined, reactive behavior becomes associated with its effects. Later, behavior is elicited by the desire to generate the associated effects again under different but related circumstances, leading to further learning (Hoffmann, 1993, 2003; Stock and Stock, 2004; Butz and Kutter, 2017). ActInf thus formalizes how internal generative structures inevitably reflect the (hidden) outside reality—or at least those components of reality that tend to affect the well-being and mere existence of the living being in question (Friston, 2009; Butz and Kutter, 2017).

The ActInf formalization subsumes three distinct, highly interactive inference processes:

1. Fast, prospective ActInf, which infers behavior from projections into the future, aiming at minimizing expected free energy, which quantifies both expected uncertainty as well as homeostasis.
2. Fast, retrospective inference to adapt current model activities to the gathered (attended-to) sensory information and the hidden causes, which (as the brain's generative model believes) seem to explain away the sensory information (including, for example, the behavior of others, physical objects, etc.).
3. Much slower, longer-term, consolidating inference, which refines, modifies, and extends the brain's generative model itself, that is, its internal predictive encoding structures. Due to the free energy formalization, the process will focus learning on those experienced aspects, episodes, events, and components thereof that appear relevant to decrease (future) free energy.

In the REPI model, we focus on simulating the first two processes, providing presumably learned predictive encoding structures as the available generative model.

Since the reminder of this work focuses on the inference of behavior and involved effort, a formalization of prospective ActInf will be useful. To do so, it is necessary to specify the to-be-minimized free energy (Friston et al., 2015b, 2018; Gumbsch et al., 2021a). Given a particular policy π, which generates actions *a* in the light of the current belief state *b*_*t*_ and system needs *n*_*t*_ considering a particular, possibly adaptive, temporal horizon τ = {*t, t* + 1, *t* + 2, …, *t* + *T*} with depth *T*, the anticipated free energy when pursuing policy π can be written as:


(1) $$\begin{array}{l} \begin{array}{c} \hat{\text{FE}}\left(\pi ,\tau ,b_{t},n_{t}\right)=\underset{\text{predicted divergence from desired states}}{\underset{︸}{\text{KL}\left[Q\left(o_{\tau }\;|\;b_{t},\pi )||\;P(o_{\tau }\;|\;b_{t},n_{t}\right)\right]}}\\ \;+\underset{\text{predicted uncertainty}}{\underset{︸}{{\text{E}}_{Q\left(b_{\tau }|b_{t},\pi \right)}\left[\text{H}\left[P\left(o_{\tau }\;|\;b_{\tau }\right)\right]\right]}} \end{array}, \end{array}$$

where prior densities are denoted by *P* while expected posterior densities are denoted by *Q*. The KL divergence is calculated as the expected divergence of posterior observation densities, when following policy π starting from belief state *b*_*t*_, from the prior desired observation densities *P*(*o*_τ_ | *b*_*t*_, *n*_*t*_) over the temporal horizon τ. Meanwhile, predicted uncertainty quantifies the entropy H over expected future observations dependent on expected policy-dependent future belief states E_*Q*(_*b*__τ_|*b*_*t*_, π)_, when following policy π starting from belief state *b*_*t*_.

Driven by the purpose to minimize free energy, and thus to maintain internal structure, Equation (1) essentially quantifies a payoff, which may be equated with the decrease in free energy compared to the possible development of free energy given inactivity.

### 3.2. Information Processing Cost

Meanwhile, though, invoking activities and focus will come at a cost, which has been equated with the change from a prior to a posterior distribution in terms of KL divergences (Ortega and Braun, 2013; Genewein et al., 2015; Zenon et al., 2019):


(2) $$\begin{array}{l} C\left(P,Q\right)=\text{KL}\left[Q\left(x|e\right)||P\left(x\right)\right], \end{array}$$

which quantifies the investment cost of changing a prior probability density over some representational generally continuous space *x* ∈ ℝ into a posterior distribution over this space given a source of evidence *e*.

When furthermore assuming a density *E* over these evidences—be they sensory information about body or environment or also internal model expectations or motor activity—a more accurate cost estimate takes the weighted mean over the possible evidences, yielding a form of mutual information:


(3) $$\begin{array}{l} C\left(P,Q,E\right)=\underset{e\in E}{\sum }p\left(e\right)\text{KL}\left[Q\left(x|e\right)||P\left(x\right)\right]={\text{I}}_{Q}\left(E,P\right). \end{array}$$

According to this equation, the cognitive effort to direct behavior and thoughts toward desired states and away from expected observational uncertainty (cf. Equation 1) can be equated with the mutual information I_*Q*_ between the state-informative evidence density *E*, which we here assume to be discrete for simplicity reasons, and state estimation prior *P*(*x*), given the conditional model *Q*, which maps evidence onto posterior state estimations.

The system then ideally finds the optimal behavioral policy π^*^ that minimizes anticipated cognitive costs, that is, information investment costs, and anticipated free energy. When the gain in free energy minimization is larger than the cognitive costs, the effort is worth its while. Effort costs and utility gains thus need to be well-balanced. Accordingly, a weighting factor has been introduced as a Lagrangian multiplier that factors the optimization cost into the expected utility gain (Ortega and Braun, 2013), allowing an adaption in how much cognitive investment is currently worth the while. This factor may essentially be related to the current cognitive resources available to an agent, which is related to inner homeostasis and thus intrinsically part of Equation 1. In the future, the fixed horizon in Equation 1 may thus be subsumed by a dynamic horizon that may optimally take planning costs and available resources into account (cf. Gumbsch et al. 2021a for a similar proposal). For now, a simplifying alternative to balancing the two aspects lies in focusing on solving one particular task and assuming that maximal available cognitive effort is spent on solving it at any current point in time. In this way, cognitive costs dominate behavioral and cognitive processing and decision making: the larger the needed density changes to process a task, the slower the behavioral decision making.

To summarize: there is compelling evidence that our brain is developing generative predictive model(s) by means of ActInf. Concurrently, the developing models enable ActInf to focus our computational resources on anticipated needs. Combined with information processing cost considerations, ActInf thus controls how computational resources are invested, considering the future in the light of the present, the current needs, and our so-far gained behavioral and model-predictive knowledge. It continuously adapts the currently active internal models in the light of the accumulating evidence, develops these models further over time, and determines current attentional dynamics, reasoning, decision making, and behavioral control by attempting to minimize expected free energy (Equation 1).

The biggest criticism on ActInf and the information cost formalization from the cognitive science side lies in the testability and falsifiability of the involved formalism. This is particularly the case because the involved generative model is not specified. Indeed, ActInf may play out not only during ontogenetic development and the hear-and-now, but even on evolutionary time-scales, creating particularly well-suited bodily properties (such as our hands), developmental pathways (such as our brains' modular structure), and consequent inductive learning biases (Pfeifer and Bongard, 2006; Butz and Kutter, 2017)—all influencing generative model development. We thus now scrutinize likely properties of the developing generative model.

## 4. Event-Predictive Structures

EPCog emphasizes that event-predictive encodings are particularly prominent in our brain, as suggested from various disciplinary and interdisciplinary perspectives (Butz et al., 2021). Coming from the developmental psychology side, for example, Dare Baldwin and Jessica Kosie emphasize that events are inferred from our sensorimotor experiences:

“Events–the experiences we think we are having and recall having had—are constructed; they are not what actually occurs. What occurs is ongoing dynamic, multidimensional, sensory flow, which is somehow transformed via psychological processes into structured, describable, memorable units of experience.” (p.79, Baldwin and Kosie, 2021)

In relation to ActInf, we appear to learn to construct progressively more abstract event-predictive structures in a self-motivated manner. Over time, we even learn to express some of these structures via language. From an evolutionary, cognitive effort-oriented perspective, it may be said that over the course of our lifetime our brain attempts to minimize its cognitive effort to life a “successful” life (ultimately from an evolutionary perspective Darwin 1859; Dawkins 1976). To succeed in our challenging social and cultural cooperative and competitive world, it appears that evolution has given our brains the tendency, or inductive learning bias, to compress our experiences into abstract, conceptual, symbolizable structures (Deacon, 1997; Butz and Kutter, 2017).

In behavioral psychological research, *event-files* have been characterized as behavioral units that commonly encode actions with their consequences, allowing the triggering of actions by their anticipated effects (Hommel et al., 2001). In more observation-oriented event segmentation studies, strong commonalities have been identified in segmenting movies into event units at various levels of granularity (Zacks and Tversky, 2001; Zacks et al., 2007). Meanwhile, memory research has shown that events are memorized as units of experience, while event boundaries characterize predictably unpredictable decisions, for example, of what a person is going to do next after having finished a particular (sub-)task (Baldwin and Kosie, 2021; Kuperberg, 2021). On the language level, events constitute a critical structural component not only in research on grammar and semantics, but also in studies on metaphors and analogies (Lakoff and Johnson, 1980; Gentner and Markman, 1998; Pietroski, 2000; Gehrke and McNally, 2019; Ünal et al., 2021). Encompassing cognitive theories have also used the event concept extensively, such as the theory of conceptual spaces and the geometry of meaning (Gärdenfors, 2000, 2014).

Recent more explicit theories on EPCog have proposed that events consist of multiple, event-characterizing components (Butz, 2016; Butz et al., 2021):

- actions as well as other forces, which dynamically influence the event dynamics;
- entities involved in the event including their roles (e.g., agent, recipient, tool) and other critical properties (e.g., agentiveness, material properties);
- spatiotemporal relations and dynamics between the involved entities while the event unfolds;
- when agents are involved, intentions that trigger particular behavior.

These components need to be flexibly bound into current events, which are then characterized and constituted by their components and their interactions. The required binding process appears to be biochemically effortful, as it requires the selective synchronization of various brain areas, effectively binding the involved components and spatiotemporal dynamics into event structures (Engel et al., 2001; Buckner and Carroll, 2007; Fries, 2015; Daume et al., 2017; Misselhorn et al., 2019; Frings et al., 2020; Stawarczyk et al., 2021).

Events can be closely related to scripts in traditional cognitive science. Moreover, research on schema structures is closely related. In these cases, though, mostly linear, well-ordered, and often fully symbolic structures were assumed. Events seem to be more flexible than this, as recently highlighted from various research perspectives (Elman and McRae, 2019; Baldwin and Kosie, 2021; Butz et al., 2021; Kuperberg, 2021; McRae et al., 2021). That is, the dynamic construction and activation of events can unfold in highly varying manners. On the one hand, it is stimulated bottom-up by the outside environment while inferring the currently activated internal generative model. On the other hand, it is controlled top-down, driven by current and anticipated, task-oriented, bodily, motivational, and emotional needs. The dynamic and selective activations, driven by the ActInf principle, essentially correspond to the dynamic consideration of the following conceptual questions: Which behaviorally relevant entities are present and which events dynamically unfold in the outside environment? Which behavioral and other counterfactual alternatives should be considered? Which dynamical interaction consequences may be relevant?

To be able to encode more complex event and event progressions, scene embeddings offer additional support. The ActInf of critical scene-characterizing properties, in which currently considered events are embedded, may constitute a hallmark of our imagination. We all know this rather well when reading a fiction book. If the book is good and we are in the right mind set, our imagination creates (abstract) scenes and fills-in unmentioned aspects with most plausible components—augmenting the stories with deeper gist, background, intentions, and emotions. There is evidence that network activity dynamics unfold within a *default network*, which appears to both maintain an actual event-specific thought and coordinate switching dynamics between thoughts (Buckner and Carroll, 2007; Buckner et al., 2008; Stawarczyk et al., 2021). The REPI model, which we introduced in the following section, essentially mimics such dynamics on an admittedly still rather simple level.

## 5. Resourceful Event-Predictive Inference

REPI integrates formalizations of cognitive effort and ActInf with the EPCog perspective. That is, REPI assumes that event-predictive ActInf strives to continuously maintain and infer the currently deemed relevant event-predictive encodings about, and interactions with, the outside environment. Moreover, ActInf controls anticipatory and reflective event dynamics as well as switches between events while accounting for the involved cognitive effort. Meanwhile, ActInf will tend to maintain multiple, possibly competitive, event hypotheses in parallel, for example, when facing a complex problem with a yet unknown problem solution or when pursuing task switching.

REPI equates cognitive effort with the effort to dynamically activate precise event-predictive encodings via active inference, including retrospective, prospective, alternative, and counterfactual encodings. The effort essentially lies in adapting internal activities and choosing behavioral activities for the minimization of both task-respective divergence and observational uncertainty. Meanwhile, theories of event-predictive cognition suggest that the involved activities are constituted by interactive, event predictive codes, which predict not only the unfolding stimulus dynamics but also each others' dynamics.

Because the adaptation processes fundamentally depend on the current availability of (event-) predictive encodings, a particular task will be solved the easier the more task-concrete encoding are available. Vice versa, the more unusual a particular task is, that is, the more incompatible required task responses are to the so-far learned predictive encodings, the harder it will be to inhibit the learned encodings and to activate current task-relevant components.

### 5.1. Preparation and Processing Information Effort

Considering the effort of focusing densities in relation to EPCog, it will be effortful to selectively activate precise event-predictions, or foci, away from previous foci as well as from uncertainty and general lethargy. That is, cognitive resources are needed both to activate new foci, to deactivate previous ones, and to invoke structure away from uncertainty, that is, away from a uniform distribution (maximal entropy).

Because the ease of activating particular densities will depend on the (i.e., learned) predictive encoding structures available, the more unusual a particular event is, the more effortful it will be to encode it. Similarly, the more unusual, unexpected, or counter-intuitive the binding of individual event components is, the more effortful the binding will be. Meanwhile, inhibiting competing events as well as considering multiple alternative events will be effortful. Even more effortful will be the successive activation or parallel maintenance of mutually inhibitory encodings, such as when a stimulus needs to be mapped first onto the right response side and then onto the left response side, due to a task switch.

Since considered events may be integrated into scenes, the complexity of and the prior knowledge about a considered scene will also play crucial roles. First, the ActInf of particular scene aspects and un-mentioned but event-relevant components for the production of coherent mental content will be effortful. The more common and well-known an encoded scene, the fewer cognitive resources will be needed to activate its compressed scene-characteristic event structures. The activation of scene-unusual, or even scene-contradictory, attributes will be resource-demanding, because their activation will be inhibited by the scene code. When alternative scenes need to be considered, their compatibility or logical temporal progression will influence the effort to maintain them in parallel.

For example, the entities and interactions necessary to prepare a cup of tea can be effectively encoded because we know all involved entities and interactions well. Its encoding is even less demanding, once we have developed an even more integrative event-predictive schema, which predicts the involved sub-events, entities and conditional event progressions a priori, for example, when considering “preparing a cup of tea” (Kuperberg, 2021). Similarly, much less cognitive effort is necessary when succeeding in processing a multitude of stimuli in an integrative, Gestalt-oriented manner—as is the case when, for example, recognizing a human figure walking in a point-light motion display (Johansson, 1973; Pavlova, 2012; Sadeghi et al., 2021).

In sum, the preparation and processing of actual events and event progressions within scenes can be assumed to be the more resource demanding:

1. the more individual entities are involved,
2. the more distinct their current spatial relations and dynamics are,
3. the more distinct their properties are,
4. the less typical or well-known the respective components and interactions are,
5. the more distinct (non-)agentive roles are involved, and
6. the fewer precise prior event-predictive encodings are available for activating the content.

The activation of a particular event will also depend on previous content activations: the more the current event differs from the previous one, the more effortful the switch. Moreover, the fewer this particular event switch has been experienced in the past, the more resource-demanding it will be, because it is less expectable.

### 5.2. Cognitive Effort When Processing (Successive) Tasks

When facing a concrete task, various components of cognitive effort are involved. During task preparation, suitable event-predictive task-specific priors away from previous posteriors, latent habitual priors, and uniform entropy need to be activated. These include priors on the expected, relevant sensory stimulus information, the expected mappings—or, more generally speaking, computations—needed to transform the stimulus into an appropriate response, as well as priors over potential responses. When processing a task-informative stimulus these priors will be adapted further. For example, in a priming task the prime may bias response tendencies. Similarly, when the stimulus informs about likely upcoming actual imperative stimuli or the upcoming task, stimulus priors, stimulus-response mapping priors, and response priors will be focused further.

When processing the task-imperative stimulus then, super-threshold activities need to be reached while inhibiting incompatible priors in perception, in competing task sets, and in the action choices. This principle is closely related to dynamic neural field theory (Schoener, 2020), where sub-threshold activities in an activation field correspond to preparatory, but also latent and residual, priors. When preparation is maximally effective, expected and fully prepared stimuli will be processed fastest and with least effort, and they will yield smallest response errors. Meanwhile, when a response is overly prepared, it may be executed prematurely, or incorrectly when the imperative stimulus does not conform to the expected stimulus. This has been, for example, studied in detail in go-nogo tasks.

In the following section, concrete examples of such encodings are provided and REPI is implemented as a dynamic processing model. Model simulations yield the Simon Effect and Task Switching costs. More detailed model evaluations are needed to further verify or falsify the ability of the architecture to model more intricate task switching observations. We end with a discussion that sketches-out the potential of REPI to model other psychological experiments and typical observable behavioral effects.

## 6. Modeling Psychological Observations

The combination of EPCog with ActInf makes rather direct predictions about task-specific cognitive effort and consequent behavioral phenomena. In particular, it has immediate implications for all behavioral paradigms where multiple, including latent, stimulus-response options need to be considered and selectively activated. According to the theoretical considerations, each task setup will lead to the activation of a particular, event-predictive state of mind. ActInf will attempt to focus the available cognitive resources on the task at hand, while prior, latent activities (such as habitual stimulus-response mappings) will maintain a base-level activity of general knowledge and behavioral response structures.

In relation to expected free energy (Equation 1), the preparation to solve a particular task may be equated with desired future observations *P*(*o*_τ_ | *b*_*t*_, *n*_*t*_), which essentially quantifies the focus on the current task set. Observations here can also include motor responses and their consequences, thus triggering response-effect compatibilities (Elsner and Hommel, 2001; Kunde, 2003). Moreover, they can include the pre-activation of particular, internal stimulus-response mappings as well as the inhibition of inappropriate ones. The higher the uncertainty in this future-concerning observation density, the more difficult it will be to trigger a task-appropriate action.

Meanwhile, the second summand in Equation (1) will maintain a general alertness, aiming at decreasing general uncertainty about the environment. This component may thus lead to the generation of task-irrelevant, epistemic actions, but also to the prior activation of latent stimulus-response mappings, which have proven to be useful to decrease uncertainty—such as reacting to a particular stimulus with an orientation reflex.

### 6.1. REPI Model Implementation

**Figure 1:**
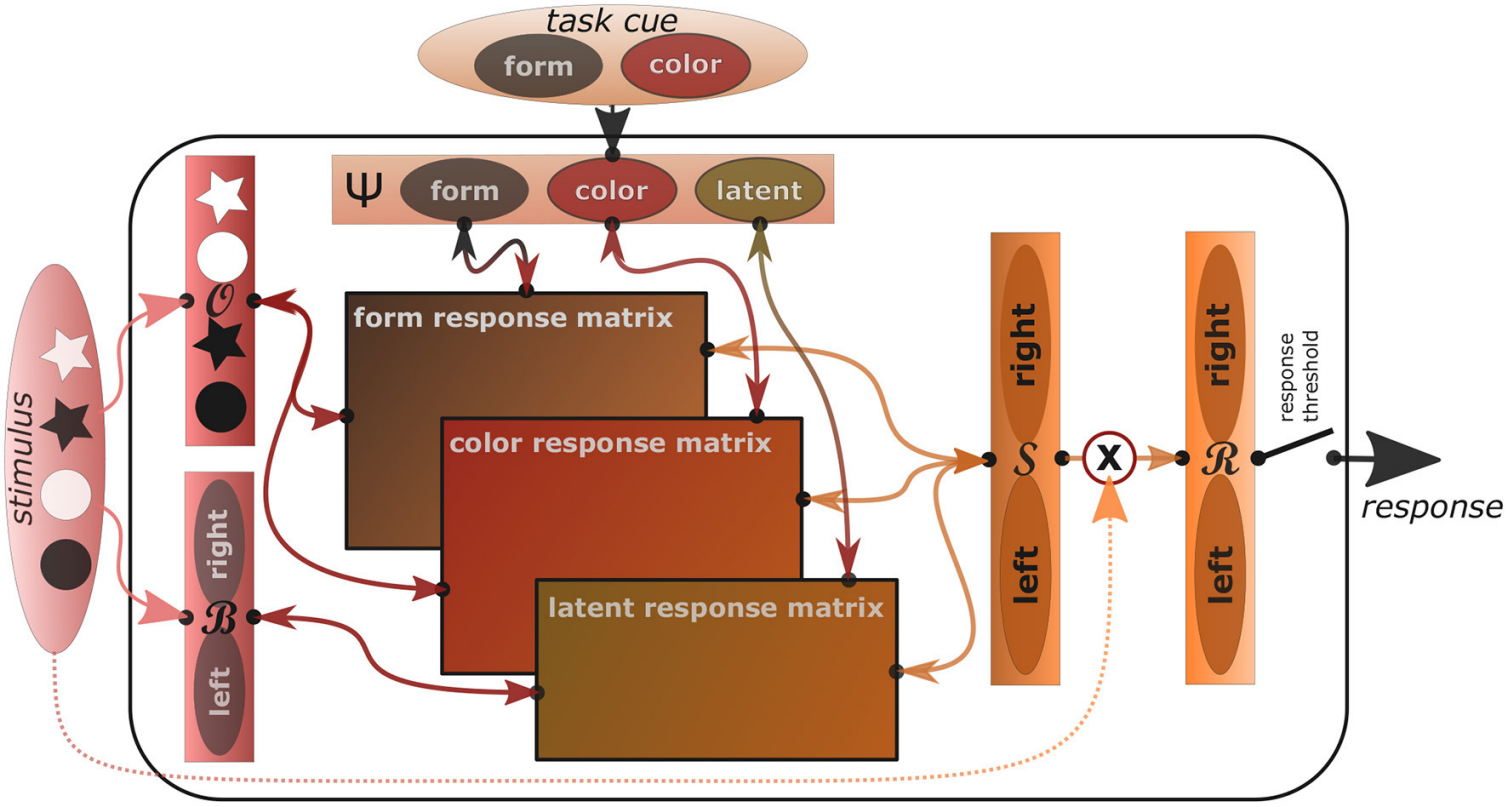
The concrete REPI model implementation is able to mimic Simon and task switching effects. It consists of five main dynamics processing modules (slim rectangles), which simulate inferences of observation densities O, observation bias densities B, task set densities Ψ, response side densities S, and actual response densities R. The wide rectangles in the center correspond to task-specific mappings from stimuli to task-respective response sides. Form- and color-response matrices map the stimulus densities onto left/right responses mixing the output relative to the form and color task activity values of the task density, while the latent response matrix adds the stimulus bias density influence weighted by the latent task activity. A response threshold determines when the response is actually triggered. Activities are only passed into the response density R upon stimulus onset. To improve comprehensibility, a particular task switching scenario is shown exemplarily, where the two tasks correspond to a form and a color task and the stimuli include black and white stars and circles, which map to either a left or right response, dependent on the task.

In order to explain the mentioned psychological behavioral phenomena not only qualitatively but quantitatively, we now introduce an actual algorithmic implementation of the REPI model. In this implementation, we focus on modeling standard tasks that elicit the Simon effect as well as task switching costs. Figure 1 shows a sketch of the task-oriented REPI model implementation, where sensory information flows into the model from the left, task-informative information from the top, response tendencies are sent toward the right, while anticipated response consequences are sent back to the left.

In all these tasks, we assume a dynamic perceptual space O, where at a point in time *t* prior densities are given by $P\left(O_{t}\right)$ and posteriors by $Q\left(O_{t}\right)$. Similarly, we denote the relevant task space by Ψ, with priors *P*(Ψ_*t*_) and posteriors *Q*(Ψ_*t*_). As an additional latent observational bias space, we model B. Moreover, we model response side dynamics in S and the actual response dynamics in R.

During a trial, these densities are dynamically adapted toward particular target values. In each iterative update step, all densities are adapted from priors to posteriors, which constitute the priors in the next time step. The update is computed as follows:


(4) $$\begin{array}{l} Q\left(X_{t}\right)\propto P\left(X_{t}\right)+\alpha \epsilon_{t}G\left(X_{t}\right), \end{array}$$

where α denotes a rate factor, ϵ_*t*_ the currently available cognitive resources per time step (e.g., per second), X∈{O,B,Ψ,S,R} denotes the density that is adapted, and $G\left(X_{t}\right)$ denotes the current targeted density, that is, the ideal goal state REPI strives for via active inference. In the simulations below we set the simulation speed to 10 ms per iteration and thus α = 0.01, while we set the resource strength to ϵ = 6.

When focusing on one particular task, the task density *P*(Ψ) will strive to adapt toward a fully focused density, which would, for example, correspond to (Ψ|ψ_*t*_ = 1) = [1, 0, …, 0] in the case of a discrete one-hot encoding when focusing on the first task. While preparing for a particular task, or a set of tasks, the task densities will adapt to the average mixture of each possible task. Finally, to model a latent readiness to process stimuli habitually, a general latent readiness may be modeled with an offset factor λ, such that, for example, a maximal readiness for processing two equally probable tasks would correspond to:


(5) $$\begin{array}{l} G\left(\text{Ψ}|\psi_{t}\in \left\{1,2\right\}\right)=\left[\left(1-\lambda \right)/2,\left(1-\lambda \right)/2,0,\ldots ,0,\lambda \right], \end{array}$$

where we assume additional dormant tasks in the middle with zero activity.

The observational density, which focuses on processing the imperative stimulus, will generate task-particular, top-down, goal-directed stimulus expectation densities $G\left(O_{t}|P\left({\text{Ψ}}_{t}\right)\right)$ dependent on the current task density *Q*(Ψ) during the stimulus preparatory phase. Once the stimulus is presented, though, processing will switch to bottom-up evidence accumulation by setting $G\left(O_{t}|o_{t}\right)$ to, for example, a one-hot encoding of the discrete stimulus signal. While information fusion options may be included here, at the moment the implementation sets $G\left(O_{t}\right)$ either to the top-down or the bottom-up observational goal, dependent on the current stimulus availability. Meanwhile, potential stimulus biases may be processed within the bias space B, where no stimulus side bias corresponds to $G\left(B_{t}\right)=\left[0.5,0.5\right]$. The response side density is continuously updated task- and observation-estimate dependently. That is, $G\left(S_{t}\right)$ is set to $P\left(S|Q\left(O_{t}\right),Q\left({\text{Ψ}}_{t}\right)\right)$, where the respective task sets are implemented by means of task-respective mapping matrices, which map individual objects to task-corresponding response sides. Finally, once a stimulus has been perceived, the actual response density is adapted away from a uniform response density toward the current processing side density, that is, $G\left(R_{t}\right)$ is set to $Q\left(S_{t}\right)$. The actual response is then triggered once a particular response threshold θ_*r*_, which we typically set to 0.9, is reached.

Assuming, as specified above, that the full focus will lie on solving tasks during an experiment appropriately, the costs for solving a particular trial may then be quantified by the dynamically unfolding mutual information between current response tendencies and desired response tendencies given the beliefs in the current task and the current stimulus situation, that is:


(6) $$\begin{array}{l} C\left(\text{task}\right)=C\left(P\left(S_{t}\right),P\left({\text{Ψ}}_{t}\right),P\left(O_{t}\right)\right)\\ \;=\underset{\psi \in {\text{Ψ}}_{t},o\in O_{t}}{\sum }p\left(\psi \right)p\left(o\right)\text{KL}\left(Q\left(S_{t}|\psi ,o\right)||P\left(S_{t}\right)\right)\\ \;=\underset{\psi \in {\text{Ψ}}_{t},o\in O_{t}}{\sum }p\left(\psi \right)p\left(o\right)\text{KL}\left(G\left(S_{t}\right)||P\left(S_{t}\right)\right)\\ \;=I\left(P\left({\text{Ψ}}_{t},O_{t}\right);P\left(S_{t}\right)\right). \end{array}$$

In the future, several advancements in this cost computation and the distribution of cognitive resources are imaginable. These include (i) the computation and integration of costs based on mutual information in the other spaces, including the task and observational spaces, (ii) the adaptive, non-uniform, active-inference-driven distribution of computational resources over the considered density spaces, that is, adapting ϵ_*t*_ in Equation (4).

### 6.2. Modeling of the Simon Effect

The Simon effect characterizes the effect that participants respond to a stimulus faster with their ipsilateral than with their contralateral effector even when the position of the stimulus presentation is irrelevant for the actual task (Erlhagen and Schöner, 2002; Cho and Proctor, 2010). From the event-predictive ActInf perspective, paradigms that study the Simon effect essentially study the presence of latent, spatial stimulus-response mappings and their latent activity strength relative to the task-specific activities, participants attempt to focus on. The latent response mapping corresponds to an ipsilateral response to lateral stimuli, such as by an overt eye saccade, by a covert direction of attention, or by an actual manual interaction—where in the last case handedness plays an additional important role. For example, we respond to a visual stimulus by looking at it, by touching it (e.g., a power switch), avoiding it (e.g., an insect), or actively pursuing interactions with it (e.g., when washing our hands or grasping a mug). As a result, ipsilateral, stimulus-response mappings, which are predictive by nature, are latently active in our minds, facilitating many everyday interactions. In a particular experiment that elicits the Simon effect, though, these latent mappings get in the way and are hard to fully inhibit.

To model the dynamics and estimate the relative effort to solve individual trials, we ran REPI on a simulation of a typical Simon effect-eliciting task. We assumed that the task was to respond to the form of an object, ignoring its color as well as its location on the screen. Objects were simulated to be presented either centrally to the right or to the left of the center. The trial started with the presentation of a fixation cross, followed by the imperative stimulus 500 ms afterwards. The inter trial interval was set to 100 ms.

In our REPI implementation, we used the general setup shown in Figure 1 focusing on the form task and leaving the latent activity active between a value of 0.1 and 0.2, dependent on if the fixation cross or the imperative stimulus are shown or not yet, respectively. Task cue and stimulus goal signals are set to 0.9 and 0.97, respectively assuming a latent task activity of 0.1 and a slim chance to perceive one of the other objects with a probability of 0.03, respectively^1^.

**Figure 2:**
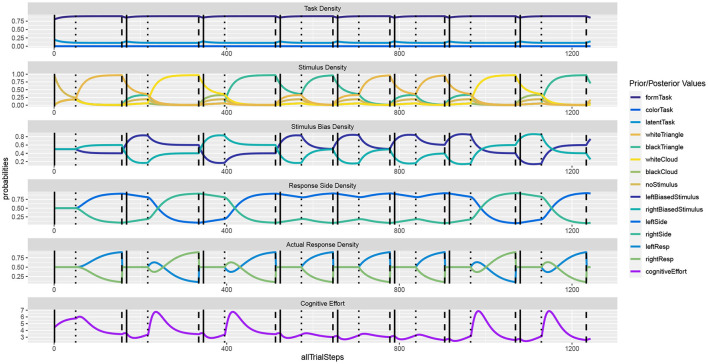
In the **Simon effect**, the response dynamics are influenced by the side of the stimulus, besides the actual stimulus identity. The dynamics clearly show how REPI gets ready for the task once the fixation cross is shown (vertical solid bars). Once the imperative stimulus is shown (vertical dotted bars), response inference is activated and evidence for either response side accumulates. The response is triggered when a particular response threshold (set to 0.9) is reached (vertical dashed bars).

**Figure 3:**
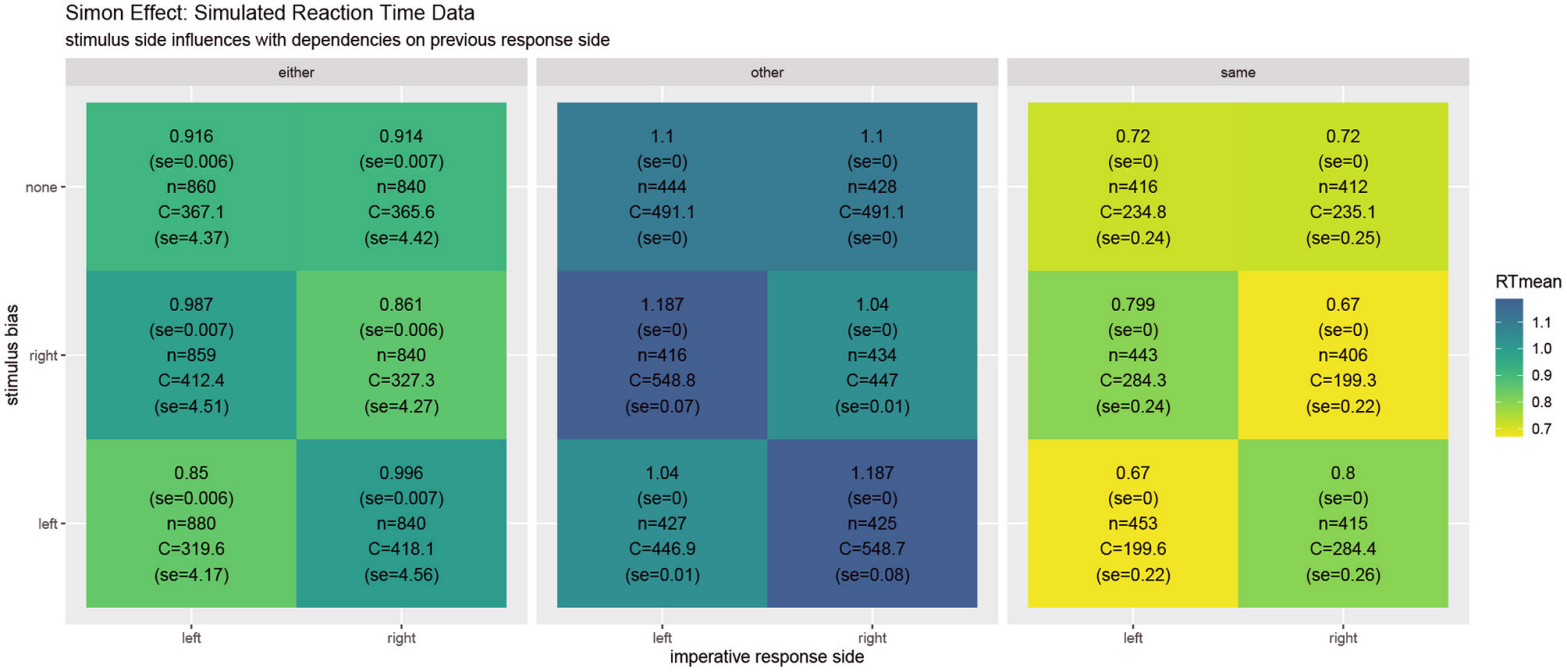
In the **Simon effect**, the reaction times clearly depend on the stimulus bias, favoring ipsilateral responses. Besides the mean reaction times, the values report standard error, the number of condition-respective trials, and mean cognitive effort costs C (task) integrated over the time from the stimulus onset until the response.

Figure 2 shows the unfolding dynamics of the simulation over the first ten trials. Moreover, the dynamics of the costs, which focuses on solving the task quickly and correctly, are plotted. Figure 3 shows the simulated reaction times as well as the resource investment determined over the time from the imperative stimulus until the response was triggered. The results clearly show the Simon effect: a stimulus that appears on the right or left is responded to faster with the compatible response side, while a stimulus in the center does not exhibit this influence. Moreover, response repetition benefits are visible, when the response side needs to be switched. The cost measurement C (cf. Equation 1) confirms that faster responses go hand-in-hand with smaller cognitive effort. Future work may also model the STROOP effect with similar principles. Here, an open question is whether modifications of the task priors will be enough to model the dominance of the word reading response in all its detail.

### 6.3. Modeling Task Switching

The task switching literature is full of additional insights on how quickly event-predictive encodings can be selectively engaged, disengaged, and inhibited. When comparing blocks with single and multiple tasks, parallel task processing costs as well as switching costs are assessed. These are needed to pre-activate the task-specific stimulus encodings and stimulus-response mappings as well as to switch between the distinct, task-specific encodings and mappings.

In contrast, mixed task blocks reveal the flexibility of the cognitive processes involved when switching between tasks (Kiesel et al., 2010; Koch et al., 2018; Frings et al., 2020). Task switching costs—that is, worse performance (typically measured in response times and response errors) when performing a different task in a subsequent trial vs. the same task—reveal that it is difficult to fully erase the task set that was relevant in the previous trial from memory. The robustness of this finding is stunning: it can be found in blocks with predictable task switches, cued switches, intermittent instructions, and even when task selection is mostly voluntary (Kiesel et al., 2010).

#### 6.3.1. Model Results

To model the dynamics and estimate the relative effort to solve individual trials, we adapted the simulation and evaluated REPI in a typical task-switching experiment. We simulated mixed task blocks, where first a cue informed about the task of the trial. After a cue-stimulus interval (set to 100 ms if not stated differently below) the imperative stimulus appeared. After the reaction was executed by REPI, the response to cue interval, which we also set to 100 ms in the reported results, commenced. We leave the other settings identical to the Simon effect simulation, except for that all stimuli were simulated to be presented centrally yielding no latent response bias. A task switch occurred with a 50% chance^2^.

**Figure 4:**
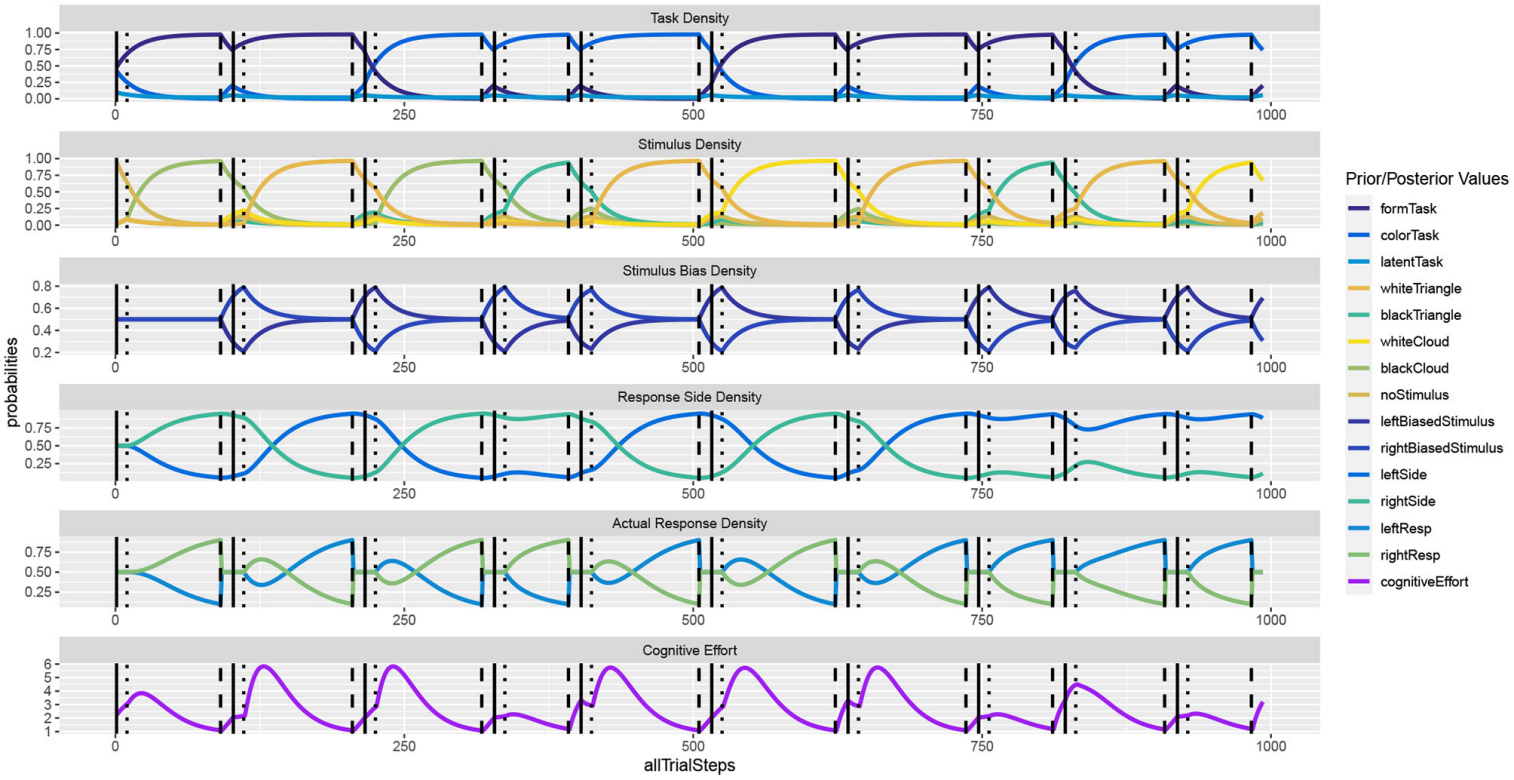
In the **task switching** simulation, the response dynamics are indirectly influenced by the previous task as well as by the previous response. Once a response is executed, response side and action densities are set back to uniform.

Figure 4 shows the unfolding dynamics of the simulation for the first 10 trials. In contrast to the Simon task, we can now observe only indirect stimulus bias density dynamics, which stem from the response side activities. Moreover, we can observe how task switches delay decision making, as do response side switches.

**Figure 5:**
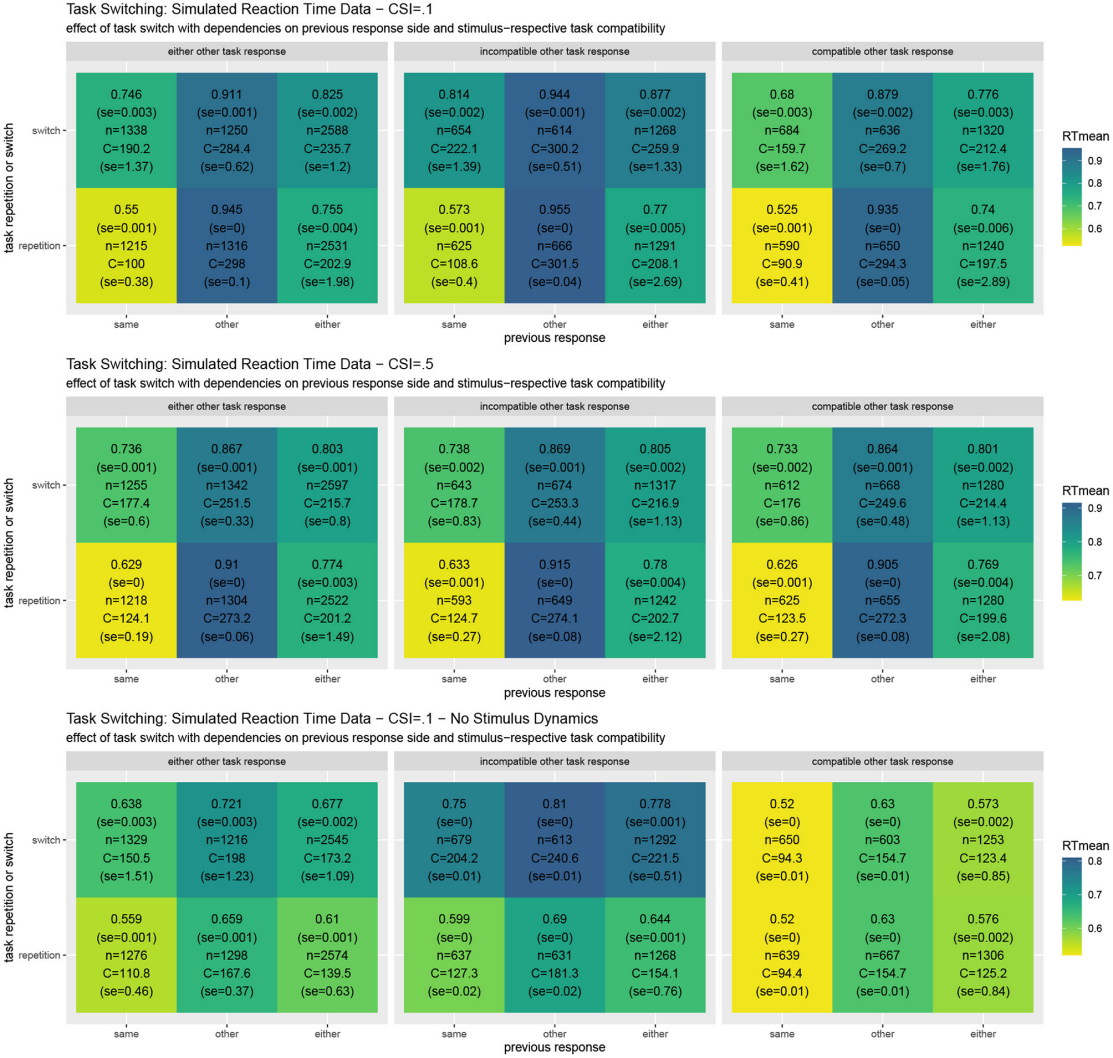
Results of **task switching** simulations averaged over twenty experiments for the full REPI model with all dynamics simulating a cue-stimulus interval of 100 ms **(top row)**. When this interval is enhanced to 500 ms **(middle row)**, the task switching effect decreases, as is well-known from results in the task-switching literature. When the stimulus density is set directly to the actual stimulus value, the predicted response distributions undergo fewer dynamical interactions **(bottom row)**. The basic REPI simulation without further parameter optimization yields reaction times that depend on whether a task switch occurred (cf. “either” column in the left block). Additionally, a strong previous response side benefit is generated, yielding faster responses for the same response side. Moreover, the compatibility of the response to the other task in a trial plays an important role (center vs. right block of reaction times). When the stimulus is assumed to be processed instantly **(bottom row)**, the expected reversal of the task-switching effect in the case of the other previous response side disappears. Besides the mean reaction times, the numbers report standard error, the number of condition-respective trials, and mean cognitive effort costs C (task) integrated over the time from the stimulus onset until the response.

Figure 5 shows simulated reaction times as well as the resource investment (cf. Equation 6). The results reveal the typical Task Switching effect as well as its decline when the response to the other task is compatible vs. when it is not. They also show that the task switching costs clearly decline with a longer cue-stimulus interval (Monsell, 2003; Altmann and Gray, 2008; Kiesel et al., 2010). Moreover, response repetition benefits are observed, which are particularly strong when the same object is presented. In comparison to the costs reported in the Simon effect simulation, it may come as a surprise that the costs for individual trials area actually slightly smaller on average. This may be the case because the costs currently only focus on the mutual information residing in the response side space. When the stimulus activities are directly set to the current value instead of dynamically adapted, the switching costs to not reverse when in the previous trial the other response side had to be chosen. This effect should be studied in further detail. When the stimulus is assumed to be processed instantly, in which case REPI acts very similar to the one in Steyvers et al. (2019), intricate interactions of the current trial situation with the previous response side disappear. Future research should further elaborate on the validity of these additional interactions. The put-forward REPI model would expect these influences, although their influences may not be as pronounced as the ones in the presented results. In fact, an instant perceptual processing essentially corresponds to very large update steps (i.e., large values of ϵ_*t*_ for the perceptual updates only; cf. Equation 4). Thus, REPI essentially subsumes the model in Steyvers et al. (2019), but, additionally, puts forward the relation to active inference, to event-predictive cognition, as well as to the involvement of cognitive effort and cognitive resources.

#### 6.3.2. Task Switching and Multitasking Literature Relations

The typical task switch results are thus explained by REPI with its implementation of the event-predictive ActInf perspective. Each task requires the activation of a particular task set, which will be encoded by corresponding event-predictive encodings, that is, the prior activation of particular stimulus-response mappings. A response is then selected based on a sufficient activity threshold, which mimics accumulator or drift models (Ratcliff, 1978; Lewandowski, 2007) and is also related to dynamic neural field approaches (Schoener, 2020). Longer reaction times thus are generated when response alternatives still have larger prior activities from previous trials. Moreover, the activity of the same response in the previous trial yields response delays in the case of a task switch, because a now incorrect stimulus-response mapping is still co-activated.

Meanwhile, an independent congruency effect can be observed, which yields faster responses when the stimuli afford the same response in the case of either current task. Interestingly, the latter is much less dependent on preparation time. Both effects can be explained by the failure to fully suppress previous task activities, thus affecting response time. If it was possible to remove or normalize over the effects of the particular pairs of tasks, response time activities may be understood in even better manners. Generally, though, REPI simulates how competing task structures may elicit congruent or incongruent stimulus-response mappings, causing the observable congruency effects.

In task-switching research, two-stage processing models have been developed, where the task selection and the actual response selection are analyzed separately (cf. Kiesel et al., 2010). Task selection corresponds to the effort to focus on one particular task while inhibiting all latent, incompatible mappings; that is, the adaption of prior task-set activities. This corresponds to the precision of the prior activation of desired observations, that is, sensory-to-motor mappings, in Equation (1), as well as additional latent activities (cf. Figure 4). Response selection then depends on the actual trial-specific stimulus as well as on the prior task set activities and competing activations. That is, response selection depends on both, the certainty which task should actually be performed and the certainty which response needs to be issued.

Moreover, given the imperative stimulus, the anticipation of the action consequences may include posterior self-evaluation attempts to maximize task-conform behavior (minimizing the KL divergence in Equation 1) while minimizing surprising consequences (minimizing expected entropy in Equation 1). These components are not included, yet, except after the actual response has been made. As a potential result, an imperative stimulus that is compatible with the other task but would require the opposite response in the other task will be particularly effortful to process, because the residual previous task activity leads to the prediction of an incorrect behavior, which needs to be avoided. Overall, larger uncertainties on both, although highly interactive, processing stages will thus yield response delays and larger errors.

Meanwhile, the refractory period to a full disengagement from the previous task will vary dependent on the complexity of the switch between tasks. Accordingly, preparation is faster the easier the switch. On the one hand side, switch training may facilitate switching between the involved, task-set characterizing predictive encodings. On the other hand, overlaps between the required perceptual stimulus processes and the relevant stimulus-response mappings will determine switching demands. If there is no overlap, switching will be rather easy. The higher the overlap, though, and the stronger the need to inhibit previously activated stimulus-response mappings, the higher the cognitive effort will be. When the task sets mutually inhibit each other, the switch of the task sets will be particularly effortful. This is the case because an even stronger change in the precision encoding is needed when the tasks overlap but demand incompatible responses. Task switching results have even shown that asymmetric tasks yield larger switch costs for switches to the easier task compared to switches to the harder task, suggesting that disengaging from inhibitions is more effortful than engaging into previously inhibited tasks (Kiesel et al., 2010). Further implementations of REPI may explore according effects.

## 7. Further Modeling Potential

In the previous section we have successfully modeled the Simon effect and task switching behavior, the results of which generally conform to the literature and have correctly revealed larger task effort when an irrelevant stimulus bias interferes, when a task switch had to be processed, when the response side needed to be changed, and when the other task requires an incompatible response. We furthermore have discussed additional open questions and modeling challenges. Here, several other paradigms and behavioral psychological phenomena are addressed and discussed, which may be modeled with REPI in the near future, revealing possible further potential as well as potential limitations of the put-forward event-predictive inference approach.

### 7.1. Theory of Magnitude

The theory of magnitude (ATOM) (Walsh, 2003) suggests that space, time, and quantity are encoded by a common magnitude system. From the event-predictive inference perspective, the common encoding of magnitudes makes a lot of sense: in the real world, magnitudes are closely related across modalities. For example, a louder crashing noise is created by a stronger force or by a larger object or by taking a longer fall onto the ground. Similarly, numerously more objects will send more visual signals, will be heavier in sum, will take longer to swipe away, and will be more effortful to create, compared to fewer objects of the same type. Thus, over development, it seems highly likely that predictive encodings form that cross-correlate various magnitudes with each other, setting the stage for the discovery of ATOM.

One closely related, particularly well-studied paradigm addresses the SNARC effect: spatial-numerical associations of response codes. In this case, numbers have been shown to be spatially-distributed on a magnitude axis, leading from left to right in societies with left-to-right scripts, presumably because the reading and writing direction guides from the past into the future, determining a latent temporal axis in our minds. Given a particular task set then, particular sets of numbers will be pre-activated in our minds, leading to the SNARC effect: even if the number magnitude does not matter for the response itself (e.g., deciding between even and odd), smaller/larger numbers are responded to faster with a left/right response (Wood et al., 2008). This fact, however, depends on the numbers that are currently mentally active in working memory as well as their spatial grounding. Accordingly, a working memory-based account of the SNARC effect has been proposed (Abrahamse et al., 2016), which highlights the importance of currently active mappings between space and numbers. For example, imagining numbers on a clock face grounds the numbers seven to 11 left of the numbers one to five, while this grounding is reversed when imagining the number on a ruler (Baechtold et al., 1998).

From the event-predictive inference perspective, these prior spatial-numerical activations correspond to current predictive encoding activities, which temporarily and latently associate numbers with space. As a result, and in close correlation with the Simon effect, investigations concerning the SNARC effect, and various related effects concerning number processing (van Dijck and Fias, 2011; Lohmann et al., 2018; Cipora et al., 2020), essentially investigate the strength of latent and temporary associations between space and numbers as well as the activation of particular spatial axes, dependent on the task at hand. Further, much more detailed, modeling efforts by means of REPI may shed further light on the concrete encodings involved and their processing dynamics in the respective ATOM-related paradigms and particular experimental setups.

### 7.2. Crossmodal Concruencies

As a final example for behavioral psychological experiments, results from studies on crossmodal congruency may be explained in a similar manner. Related to the SNARC effect and the theory of magnitude, in crossmodal congruency paradigms typically irrelevant distractor stimuli in a somewhat irrelevant modality systematically interfere with stimulus processing in the response-relevant modality.

One prominent approach was originally motivated by single cell recording results in monkeys. Individual neurons were shown to encode peripersonal spaces, responding to stimuli touching or appearing close by a particular part of the hand or face (Fogassi et al., 1996; Maravita et al., 2003). From an event-predictive inference perspective, again this comes as no surprise. As discussed above, cross-modal associations occur very frequently in our world. Thus, latent predictive encodings can be expected to develop that associate these respective stimuli cross-modally as well as across respective frames of reference. In fact, the rubber hand illusion (Botvinick and Cohen, 1998) as well as the disappearing hand trick (Newport and Gilpin, 2011) fall in the same category, in which case the brain attempts to infer a consistent explanation for the multisensory or sensorimotor signals the participants focus on, leading to an adaption of internal body state estimations (Ehrenfeld and Butz, 2013; Butz et al., 2014). During a typical crossmodal congruency task, on the other hand, irrelevant stimuli close to response-relevant tactile stimuli will interfere with according responses (Spence et al., 2004), indicating that visual stimuli are mapped onto tactile perceptions a priori, because the two modalities typically offer compatible signals in the real world.

Interestingly, the more recently investigated anticipatory cross-modal congruency effect (Brozzoli et al., 2010; Belardinelli et al., 2018; Lohmann et al., 2019) shows that peripersonal space around the hand is projected onto a planned grasp even before the actual action execution commences. These results essentially indicate that event-predictive inference anticipates action results, pre-activating particular goal constellations and according multisensory mappings. Interestingly, as would be expected from the event-predictive ActInf perspective, when uncertainty is high while approaching the object, the focus should lie on controlling the actual arm movement, thus temporarily not yielding a significant anticipatory crossmodal-congruency effect (Lohmann et al., 2019). A first variational implementation of the effect can be found elsewhere (Weigert et al., 2021).

Earlier studies on simpler action-effect compatibilites also fall into the same category: the effect of an action and the latent or temporarily activated compatibility with the action code itself, can lead to anticipatory cross-modal interactions (Elsner and Hommel, 2001; Kunde, 2003). On the dynamic event encoding level, studies on rotations have shown yet again related predictive encoding interactions: the processing of a rotating tactile stimulus on the palm, for example, interferes with the active rotation of a visual stimulus in a dual task paradigm (Lohmann et al., 2017). Earlier, visual perception was shown to be biased by the rotating tactile stimulus (Butz et al., 2010), indicating shared temporal dynamic encodings across modalities. We believe that REPI may indeed be used to model all of these effects—although open questions include the stimulus design choices, the actual involved predictive encodings, as well as the exact means to process the anticipated horizon and its effect on actual stimulus processing dynamics.

### 7.3. Social Interactions With Other Agents

In our social realities, we experience scenes, and events within, often with a multitude of entities and other agents involved. These other agents have their own minds. Thus, to foster effective and adaptive interactions between these agents, it is beneficial to know something about the others' minds, that is, to develop a theory of mind (ToM) (Frith and Frith, 2005). When engaging in social interactions, then, ActInf may consider the ToM of others, including their perception of one's own mind and particularly one's own personality. Accordingly, building up scenes with a multitude of interaction-critical agents becomes effortful and can be strenuous. The more diverse agents (colleagues, friends, co-workers, team-members etc.) one is willing and able to consider, the more effort will be needed. Their parallel activation without between-agent interference (in one's mind), is *per se* strenuous because it is impossible to maintain too many distinct predictive densities simultaneously—particularly seeing that each set of events about another agent contains recursive agent events.

This recursiveness with respect to social interactions enables us to pursue rational social reasoning, as formulated in the rational speech act model (Frank and Goodman, 2012; Goodman and Frank, 2016). On the other hand, it also requires much more computational resources, as others need to be co-processed in one's own mind. Accordingly, a rather large corpus of studies suggests that our brains, particularly when acting under time pressure, have a very hard time to consider the perspective of others, failing to avoid, for example, undesired ambiguous situations during conversations (Ferreira and Dell, 2000; Ferreira, 2008). Due to the highly resource-demanding challenge to maintain the perspective of an interaction partner besides ones own perspective, we often sidestep this perspective taking problem by simply assuming that others have the same state of knowledge as we do. Nonetheless, when retrospectively interpreting ones own utterance and the observed actual behavioral response of the current conversation partner(s), we are able to inversely infer aspects of their motivations, intentions, and state of knowledge (Baker et al., 2017; Liu and Spelke, 2017; Achimova et al., 2022), enabling us to learn about others.

In sum, social interactions will require additional cognitive resources because effective and adaptive social interactions with particular others will need to activate our ToM about the other. Moreover, the more complex that social interaction and the more unusual the other(s); the more effortful the ActInf-based control of these interactions will be. It remains an open challenge to shed further light on the involved processes and resource demands by means of socially-extended REPI-like models.

## 8. Conclusions

This paper has put forward that cognitive effort unfolds in our brain on multiple levels of abstraction, within scenes, and event-respectively. During wakefulness, our state of mind essentially elaborates on the accumulating evidence about the outside environment, binding and integrating them into currently active, behavioral-relevant, generative models about—and possible interactions with—the environment. Meanwhile, we selectively probe the future to minimize anticipated uncertainty and deviations from expected internal homeostasis. Moreover, we consolidate our accumulated, event-oriented experiences into our episodic memory and, over time, into procedural and associative memory structures. All these aspects are effortful, because they require the selective, precise, coordinated activation of event-predictive encodings, the co-activation of alternatives, and selective consolidations.

The proposal is generally compatible with The Binding and Retrieval in Action Control (BRAC) proposal (Frings et al., 2020), which offers explanations for task switching and related tasks. BRAC suggests two main cognitive processes: stimulus-response-effect combinations are bound into event files for task preparation and execution. Meanwhile, related event files are retrieved from previous trials. Viewed from the introduced resourceful event-precictive inference perspective REPI, event files are constituted by sets of predictive encodings (Butz, 2016), which predict sensory perceptions, sensory-to-motor mappings, actual motor responses, and consequences thereof. Retrieval from previous trials corresponds to residual as well as latent, habitual predictive encoding activities. Beyond BRAC, though, the proposed ActInf-induced effort perspective offers a computational formalism how binding and retrieval are elicited. Moreover, it suggests that cognitive effort can be equated with changes in the event-predictive densities that focus our minds onto particular tasks while inhibiting previous activities as well as latent, habitual activities.

The put-forward resourceful event-predictive inference model REPI also offers a computational explanation why particularly multitasking and task-switching is cognitively effortful and strenuous. This is the case because the maintenance of multiple event schemata, that is, the maintenance of a more complex, multi-task-specific, predictive encoding density is more effortful. This is particularly the case, when multiple, independent alternatives need to be maintained and, even more so, when the alternatives need to activate mutually inhibitory predictive encodings. On top of that, it is not only the maintenance of complex densities, but also the fast switching between task-respective response mapping alternatives—be they explanations, counterfactual reasoning considerations, or simple task mappings. Such switches require the temporary stronger, selective activation of task-specific densities, while inhibiting those from the other concurrent tasks, whereby ActInf pushes toward accomplishing the switches back-and forth between them.

The REPI implementations of tasks that elicit the Simon effect as well as of task switching experiments underline the potential of the theoretical perspective and ask for further research efforts. REPI has essentially shown how computational resources may be distributed by means of ActInf within the developing, latent event-predictive structures. However, for now the effort was distributed equally over all modules, that is, ϵ_*t*_ in Equation (4) was constant and had the same value in all modules. Additionally, neither sequential expectation effects nor fatigue have been modeled, yet. Equation (4) allows for such adaptations and could certainly be modified further by modeling task-oriented focus following the general active inference principle. A related challenge lies in identifying and in learning the actual event-predictive structures that are maximally useful to improve task performance. Moreover, the neural mechanisms that enable the selective activation and maintenance of multiple present, past, future, and social considerations should be scrutinized further. As a final result, the put-forward event-predictive ActInf-induced perspective on cognitive effort may not only be able to explain and algorithmically model a multitude of results from psychological studies, but may also offer design and training suggestions to facilitate our interactions with our increasingly complex, diverse, social, digitalized world.

## Data Availability Statement

The original contributions presented in the study are included in the article/supplementary material, further inquiries can be directed to the corresponding author.

## Author Contributions

The author confirms being the sole contributor of this work and has approved it for publication.

## Funding

This research was supported by the German Research Foundation (DFG) within Priority-Program SPP 2134-project Development of the agentive self (BU 1335/11-1, EL 253/8-1), the research training group 1808 on Ambiguity-Production and Perception, the research unit 2718 on Modal and Amodal Cognition: Functions and Interactions (BU 1335/12-1), and the Machine Learning Cluster of Excellence, funded by the Deutsche Forschungsgemeinschaft (DFG, German Research Foundation) under Germany's Excellence Strategy - EXC number 2064/1 - Project number 390727645. Finally, additional support came from the Open Access Publishing Fund of the University of Tübingen.

## Conflict of Interest

The author declares that the research was conducted in the absence of any commercial or financial relationships that could be construed as a potential conflict of interest.

## Publisher's Note

All claims expressed in this article are solely those of the authors and do not necessarily represent those of their affiliated organizations, or those of the publisher, the editors and the reviewers. Any product that may be evaluated in this article, or claim that may be made by its manufacturer, is not guaranteed or endorsed by the publisher.

## Footnotes

^1^The Simon effect model implementation is available online as an interactive shiny app: https://cognitivemodeling.shinyapps.io/shinysimoneffectsimulation/. It allows explorations of critical model parameters, random trial order dynamics, and resulting, condition-dependent reaction time values.

^2^The task switching model implementation is available online as an interactive shiny app: https://cognitivemodeling.shinyapps.io/shinytaskswitchingsimulation/, which allows further explorations of additional effects of the model parameters and the simulation setup.
